# Supplementary figures and images for: Molecular mechanisms linking peri-implantitis and type 2 diabetes mellitus revealed by transcriptomic analysis
Source: PeerJ. 2019 Jun 21;7:e7124. doi: 10.7717/peerj.7124 (PMC6590641; doi:10.7717/peerj.7124)

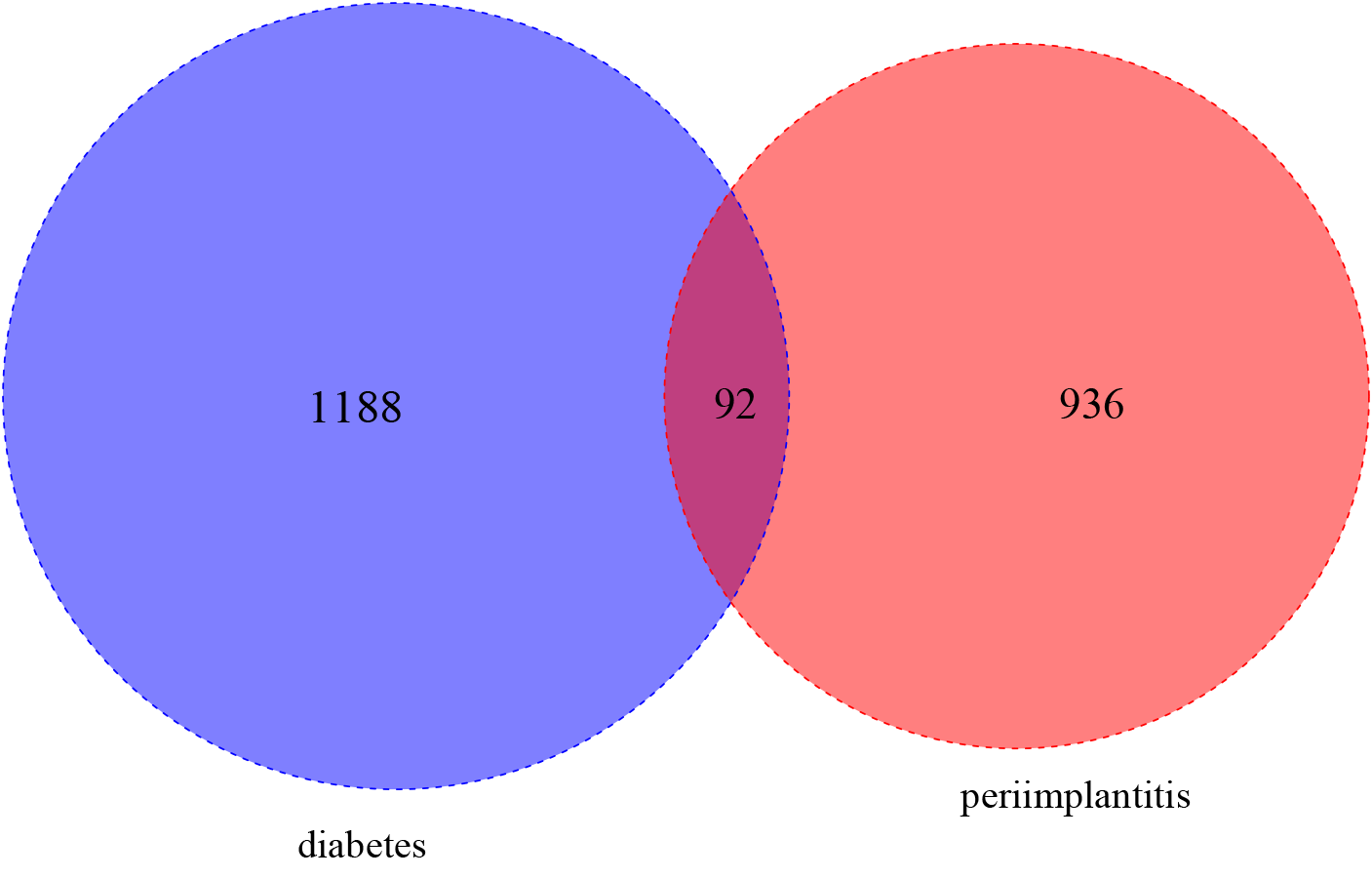

Supplement: Supplemental Information 1 [file peerj-07-7124-s001.png]

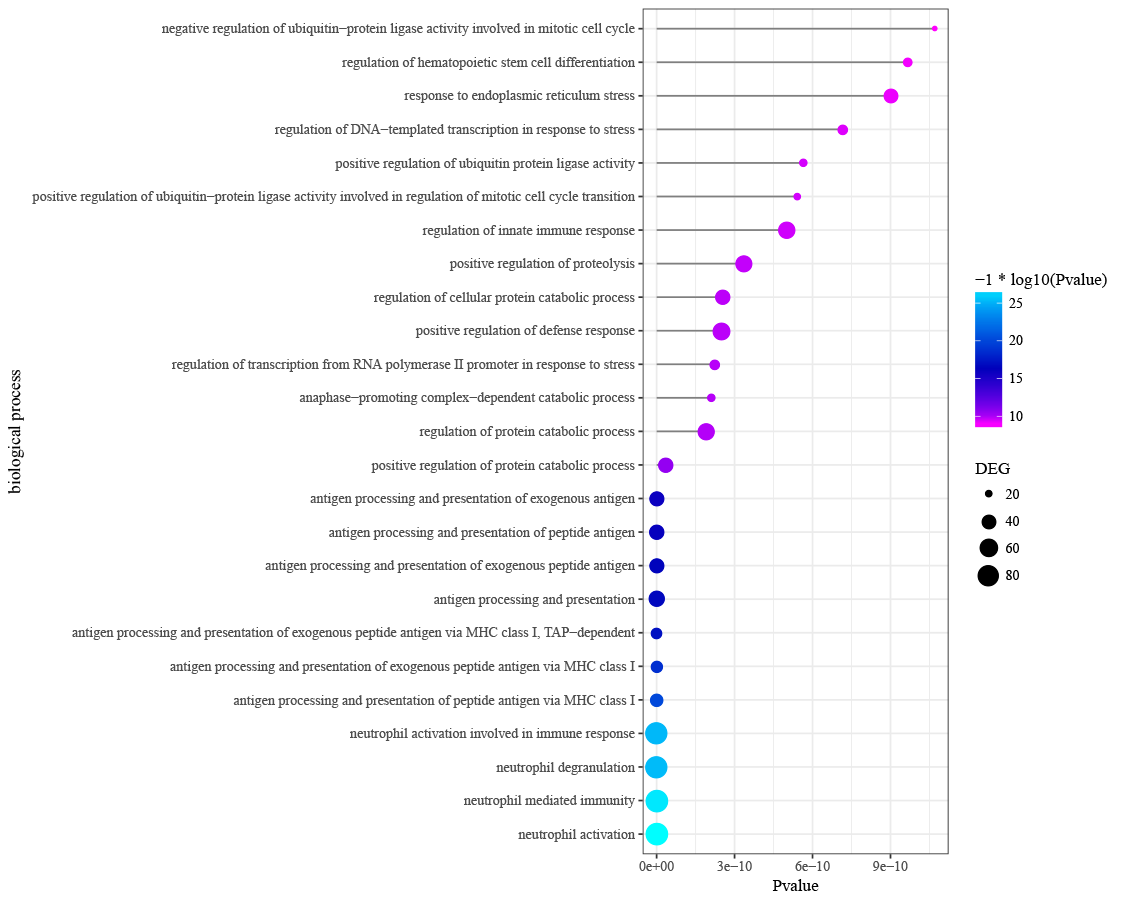

Supplement: Supplemental Information 2 [file peerj-07-7124-s002.png]

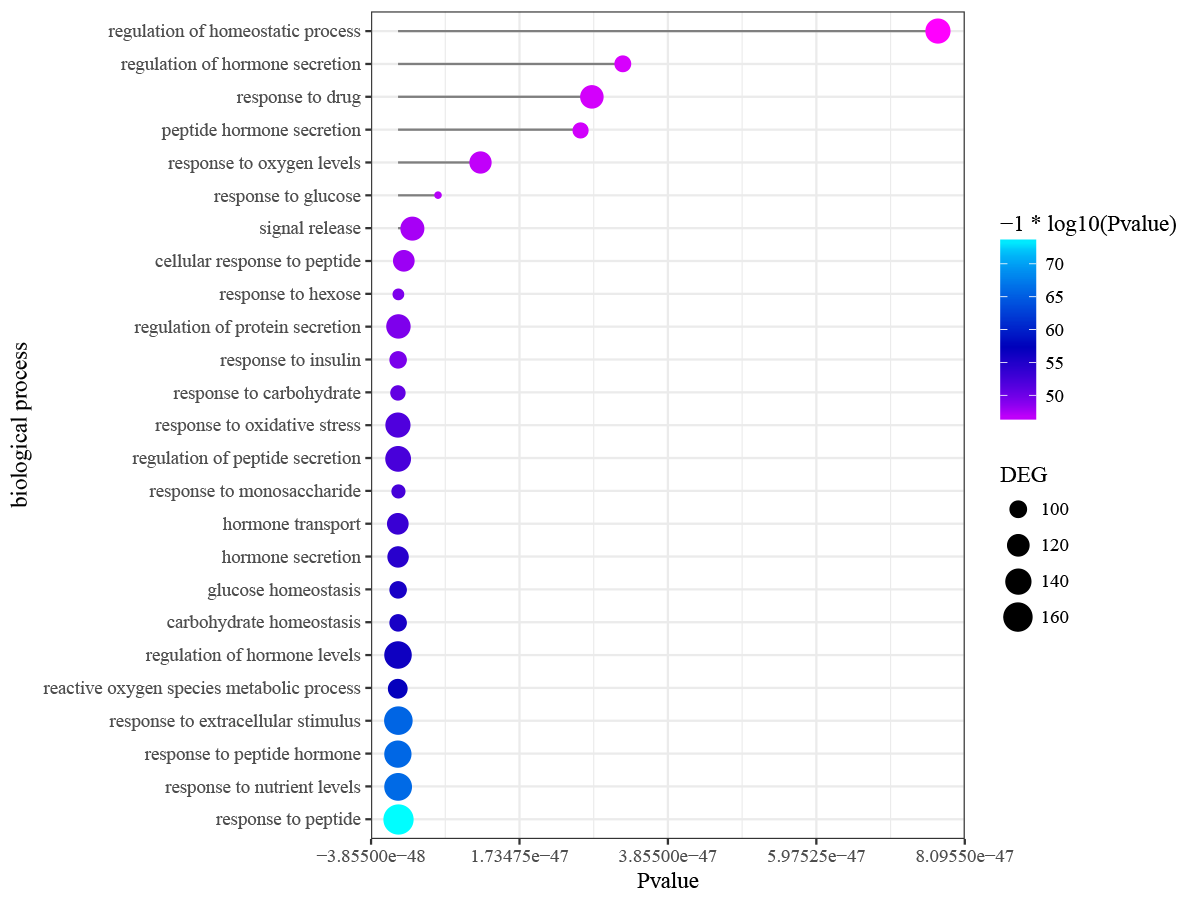

Supplement: Supplemental Information 3 [file peerj-07-7124-s003.png]

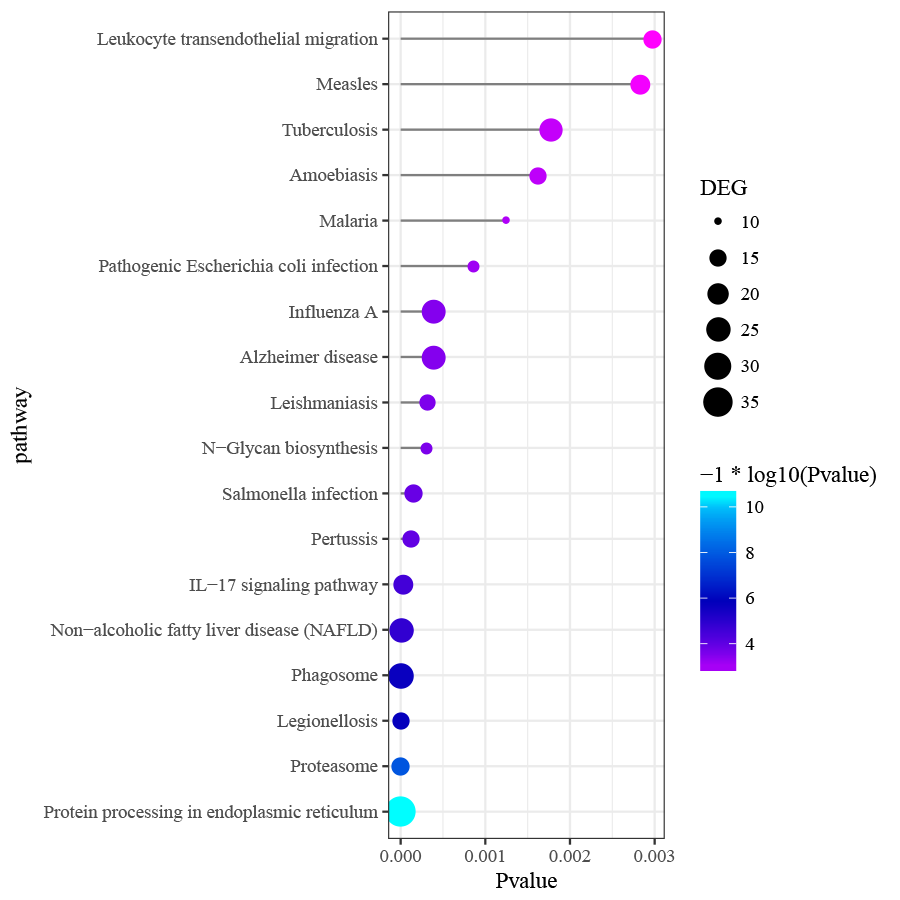

Supplement: Supplemental Information 4 [file peerj-07-7124-s004.png]

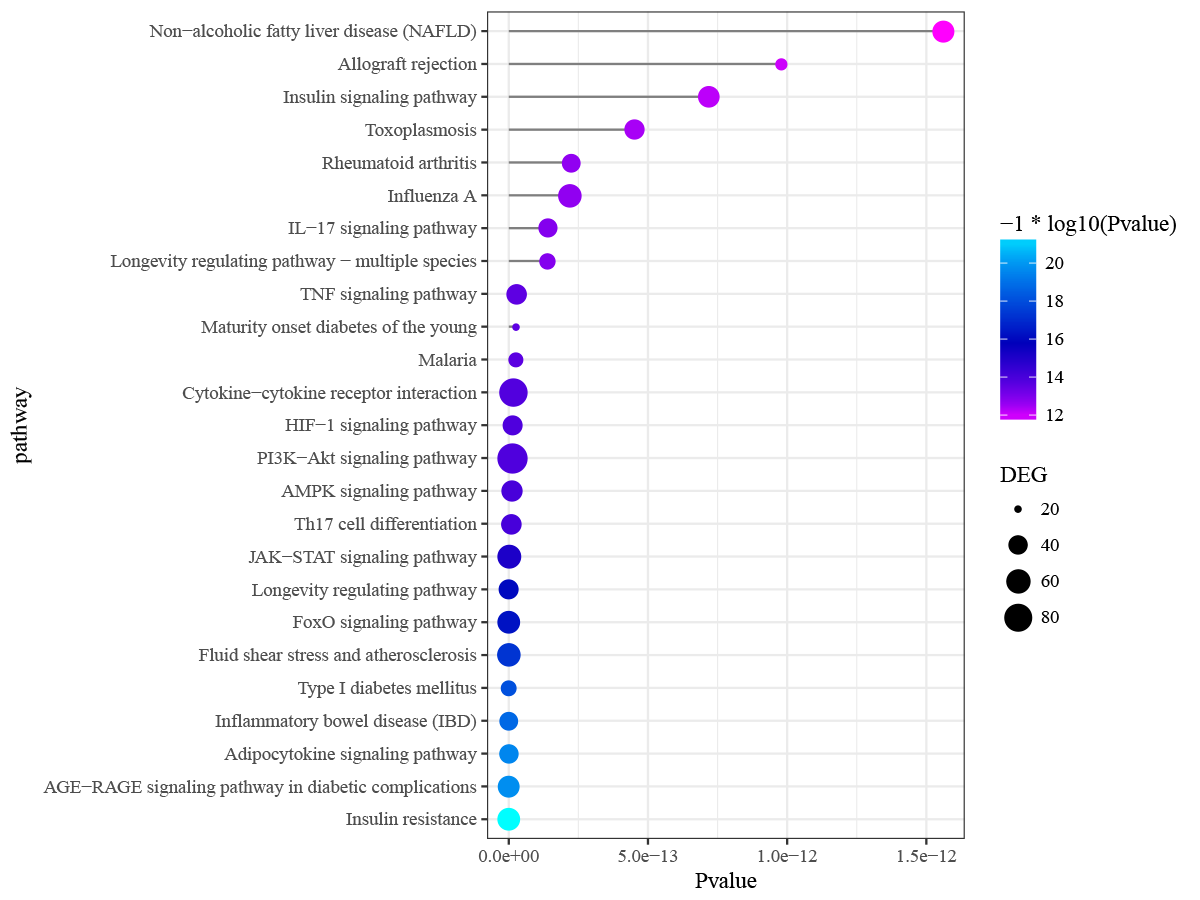

Supplement: Supplemental Information 5 [file peerj-07-7124-s005.png]

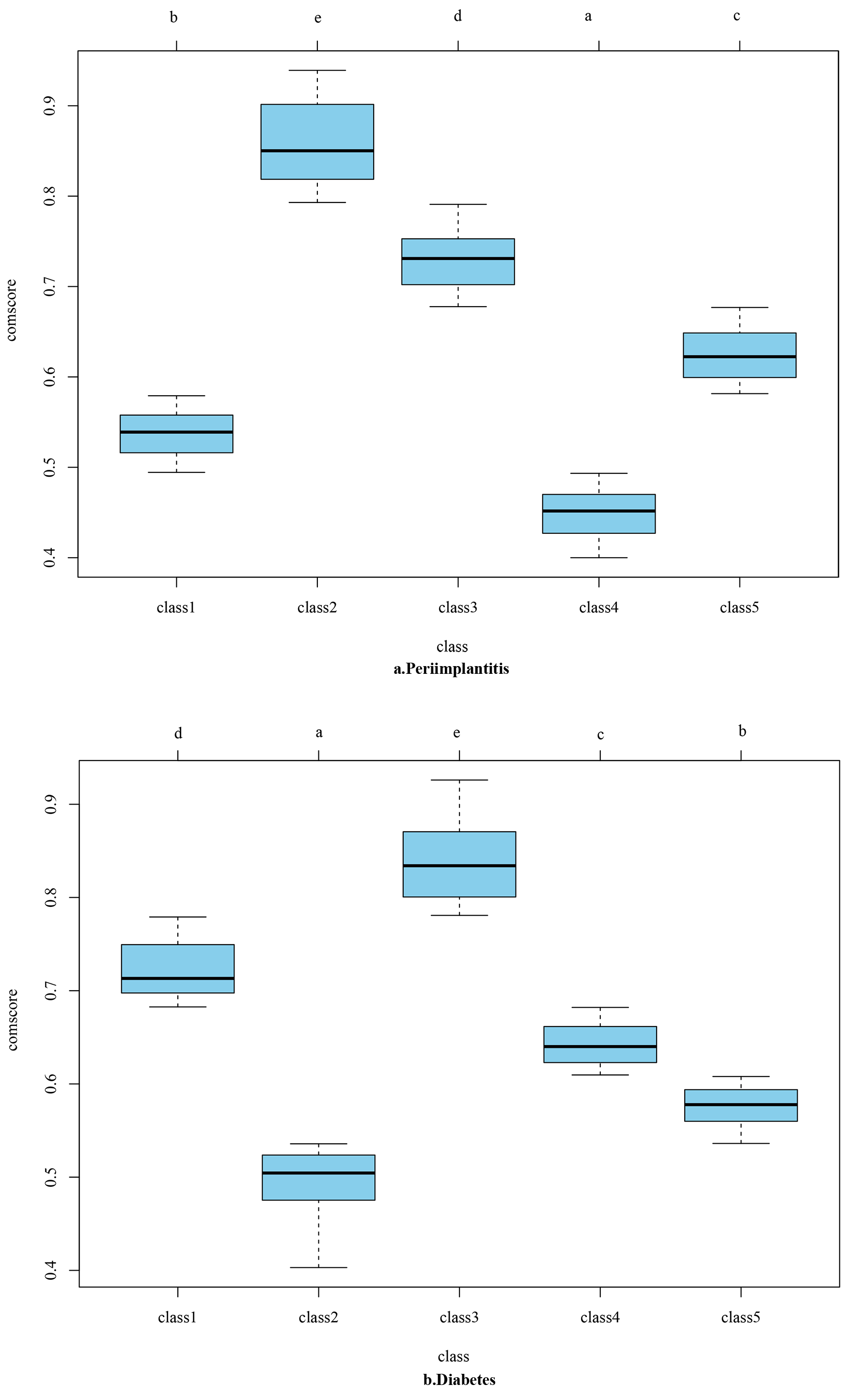

Supplement: Supplemental Information 6 [file peerj-07-7124-s006.png]
