# Supplementary material for: Molecular mechanisms linking peri-implantitis and type 2 diabetes mellitus revealed by transcriptomic analysis
Source: PeerJ. 2019 Jun 21;7:e7124. doi: 10.7717/peerj.7124 (PMC6590641; doi:10.7717/peerj.7124)
Supplement: Supplemental Information 7 — Table S1. The number of DEGs identified in two datasets (GSE33774 and GSE57631) of peri-implantitis. Table S2. Top 20 nodes in PPI networks of both peri-implantitis and T2MD. Table S3. Three leader genes shared in two selected significant classes. [file peerj-07-7124-s007.docx]

**Table S1. The number of DEGs identified in two datasets (GSE33774 and GSE57631) of peri-implantitis.**

| **datasets** | **Periimplantitis samples** | **Healthy samples** | **Platform** | **Up-regulated DEGs** | **Down-regulated DEGs** | **Total DEGs** |
| --- | --- | --- | --- | --- | --- | --- |
| **GSE33774** | 7 | 8 | GPL6244 | 158 | 66 | 224 |
| **GSE57631** | 6 | 2 | GPL15034 | 678 | 135 | 813 |

**Table S2. Top 20 nodes in PPI networks of both peri-implantitis and T2MD.**

| periimplantitis | | | | T2DM | | | |
| --- | --- | --- | --- | --- | --- | --- | --- |
| name | label | Degree | Betweenness  Centrality | name | label | Degree | Betweenness  Centrality |
| IL6 | common | 110 | 0.066558 | INS | diabete | 496 | 0.090076 |
| HSP90AA1 | common | 108 | 0.086674 | ALB | diabete | 463 | 0.066404 |
| JUN | DEG | 88 | 0.055696 | GAPDH | diabete | 415 | 0.034327 |
| IL8 |  | 77 | 0.027852 | AKT1 | diabete | 394 | 0.040254 |
| NFKB1 | common | 74 | 0.021399 | MBOAT4 | diabete | 380 | 0.025206 |
| HSPA5 | common | 73 | 0.040629 | TP53 | diabete | 378 | 0.042707 |
| CDH1 | DEG | 70 | 0.057981 | IL6 | common | 376 | 0.021643 |
| PIK3CG | common | 64 | 0.025111 | TNF | diabete | 346 | 0.021709 |
| P4HB | common | 64 | 0.019196 | VEGFA | diabete | 323 | 0.016165 |
| IL1B | common | 62 | 0.016059 | PIK3CA | diabete | 294 | 0.011573 |
| RHOA | DEG | 61 | 0.030243 | IL8 |  | 270 | 0.010011 |
| ENO1 | DEG | 58 | 0.022039 | IGF1 | diabete | 268 | 0.009889 |
| MMP9 | common | 57 | 0.016405 | MYC | diabete | 259 | 0.013882 |
| TLR4 | common | 56 | 0.014158 | EGFR | diabete | 258 | 0.01463 |
| ICAM1 | common | 55 | 0.008012 | PIK3CG | common | 253 | 0.00535 |
| TXN | common | 55 | 0.016857 | PIK3CB | diabete | 249 | 0.005612 |
| PTPRC | DEG | 53 | 0.022321 | MAPK8 | diabete | 248 | 0.01054 |
| CCT5 | DEG | 51 | 0.013908 | STAT3 | diabete | 245 | 0.009688 |
| NFKBIA | DEG | 50 | 0.008915 | PIK3CD | diabete | 242 | 0.004588 |
| PSMA6 | common | 49 | 0.012421 | NFKB1 | common | 235 | 0.005533 |

**Table S3. Three leader genes shared in two selected significant classes.**

|  | **periimplantitis** | | T2DM | | |
| --- | --- | --- | --- | --- | --- |
| **gene** | comscore_gene | cluster_gene | comscore_diabetes | | cluster_diabetes |
| **PSMD10** | 0.809333 | class2 | 0.782813 | class3 | |
| **SOS1** | 0.82892 | class2 | 0.859122 | class3 | |
| **WASF3** | 0.872375 | class2 | 0.807111 | class3 | |
